# Ring Cavity Erbium-Doped Fiber for Refractive Index Measurements

**DOI:** 10.3390/s22239315

**Published:** 2022-11-30

**Authors:** Rosa Ana Perez-Herrera, Liliana Soares, Susana Silva, Orlando Frazão

**Affiliations:** 1 Department of Electrical, Electronic and Communication Engineering, Public University of Navarra, 31006 Pamplona, Spain; 2 Institute of Smart Cities (ISC), Public University of Navarra, 31006 Pamplona, Spain; 3 INESC TEC, R. Dr. Roberto Frias, 4200-465 Porto, Portugal; 4 Faculty of Engineering, University of Porto, R. Dr. Roberto Frias, 4200-465 Porto, Portugal

**Keywords:** amplified spontaneous emission, refractive index measurements, erbium-doped fiber

## Abstract

In this study, an interrogation system based on an erbium-doped fiber ring cavity for refractive index measurements is presented and experimentally demonstrated. This cavity ring includes a 1 × 3 coupler wherein one of the fiber output ports is used to increase the optical power of the system by means of an FBG used as a reflector. The other two output ports are used as a refractive index sensing head and reference port, respectively. An experimental demonstration of this proposed sensor system for the measurement of a distinct refractive index is presented.

## 1. Introduction

The refractive index (RI) is a physical parameter of great importance in several biological and biochemical processes [1]. In recent years, RI sensing has been performed using optical fiber-based sensors due to their relevant characteristics, such as their small size (which makes them less invasive and allows RI sensing without the need for sampling), wide bandwidth, immunity to electromagnetic interference, biocompatibility, and corrosion resistance [2]. Optical fiber sensors modulated in intensity are the most suitable in practical terms for RI sensing, because wavelength-modulated sensors are generally sensitive to temperature variations [3].

Over the years, several intensity-modulated sensors have been proposed for RI sensing, namely macrobending structure sensors [4], interferometer configurations [5], surface plasmon resonance sensors [6] and others. Although these sensors have shown good sensitivity in RI sensing, they all have some disadvantage in common, namely the detection units need to be fully immersed in the medium under analysis, which, in real applications, turns out to be quite difficult and results in sensor damage, causing economic losses for the user.

To solve this problem, an alternative solution is to use fiber tips as a sensing element [7]. In this way, only the ends of the fiber tip are immersed in the medium under analysis, which reduces the probability of damaging the sensor unit, allows minimally invasive IR measurements and facilitates the easy replacement of the sensor in case of damage.

There are different fiber tip configurations for RI sensing with high sensitivity, repeatability, and accuracy. They range from the simplest, such as cleaved multimode fiber tips, to the most complex, such as microspheres [8]. Intensity sensors require signal referencing to eliminate source fluctuations and sometimes there is high signal noise when the sensors are immersed in liquids [9,10]. On the other hand, ring cavity erbium-doped fiber lasers (EDFRLs) use the gain provided by the erbium-doped fiber (EDF) more efficiently, and they also have a cavity free spectral range (FSR) that is twice as large for the same cavity length, compared with linear cavity lasers.

In this study, an interrogation system based on a fiber ring cavity design with a 1 × 3 optical coupler and assisted by an FBG reflector is proposed. In this configuration, one of the fiber output ports is used to increase the optical power of the system by means of an FBG, and the other two output ports are used for RI measurements and the reference port, respectively. A study of the measurement of different RIs is presented.

## 2. Experimental Setup

**Figure 1:**
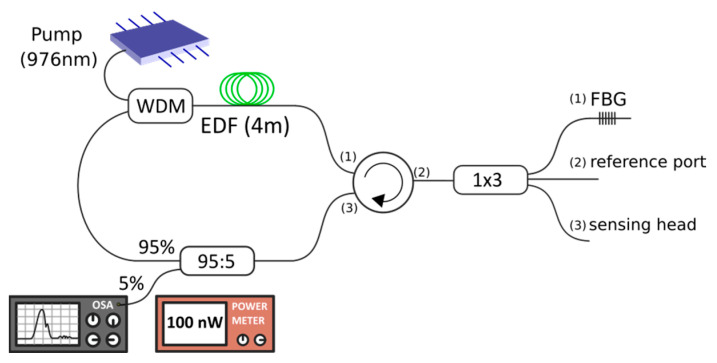
Experimental setup of the ring cavity EDF used for refractive-index measurements.

The experimental setup of the proposed ring cavity erbium-doped fiber (RCEDF) is shown in Figure 1. As illustrated, a 980/1550 nm wavelength division multiplexer (WDM) is used to inject 20 mW at 976 nm pump power into the RCEDF. This laser diode is temperature controlled to ensure that the laser output is as stable as possible. The gain medium is connected to the common port of the WDM and it consists of 4 m of highly doped EDF followed by a 3-ports optical circulator, in which port 2 is connected to a 1 × 3 optical coupler. The first of the output ports of this coupler is connected to an FBG centered at 1543.7 nm at the end of which a cross section is made. The other two output ports of the coupler are also cross-cut. One of them is used as a reference and the other as a sensing head. Finally, a 95% coupler is used in order to extract 5% of the signal from the ring to an optical spectrum analyzer (OSA) or a power meter. This configuration, based on circulators, ensures unidirectional operation and therefore avoids spatial hole-burning (SHB). All the experimental measurements were conducted at room temperature and no vibration isolation or temperature compensation techniques were employed.

## 3. Results

### 3.1. Output Spectrum

**Figure 2:**
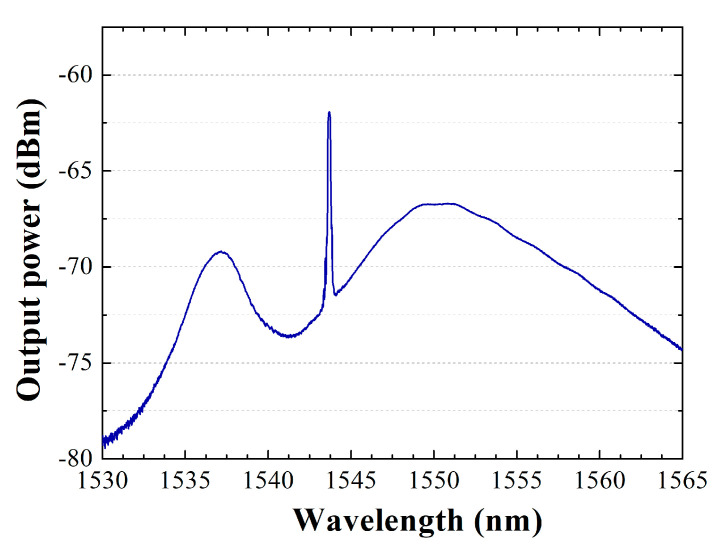
RCEDF output spectrum pumped by a 976−nm laser at 20 mW, when both output port 2 (reference port) and output port 3 (sensing head) are in contact with air.

Figure 2 shows the RCEDF output spectrum when pumped by a 976-nm laser at 20 mW, and the reference and sensing head are in contact with air. As expected, the laser condition was not reached due to the small amount of pump power, and amplified spontaneous emission is obtained by using this cavity ring configuration. The low pumping power level introduced in this interrogation system results in the stimulated emission state not being reached and, as can be seen in Figure 2, the characteristic curve of the ASE appears; its shape will largely depend on the type of erbium-doped fiber used as gain medium. As indicated in the introduction of this study, and will be experimentally demonstrated in the following sections, the use of an FBG reflector, acting as an optical filter, achieves an increase in received optical power of the measurements made and reduces the losses introduced in the interrogation scheme.

The measured output spectrum when pumped by a 976-nm laser at 20 mW shows an output peak power, centered on the Bragg wavelength of the reflector, at 1543.7 nm, of around −62 dBm, with an optical signal to noise ratio (OSNR) as low as 10 dB.

### 3.2. Fiber Ring Cavity Characterization

After spectral analysis of the ring cavity, a refractive index characterization is performed by immersing two of the coupler output ports in air, water, or ethanol. For this purpose, and with the use of the power meter, the losses produced in each of these situations are evaluated.

**Figure 3:**
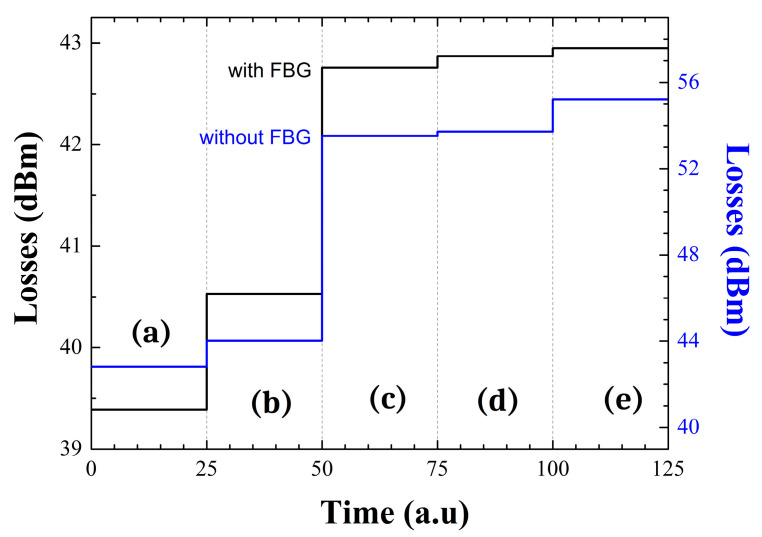
Sensor characterization with (black line) and without (blue line) FBG when: (**a**) both reference and sensing head are in contact with air; (**b**) the reference is immersed in water and the sensor remains in air; (**c**) both are submerged in water; (**d**) reference port-end and sensing head are in contact with ethanol and water in that order; (**e**) both ports are immersed in ethanol.

Figure 3 shows a comparison between the results obtained when (a) both the reference and sensor are in contact with air, (b) the reference is immersed in water and the sensor remained in air, (c) both are immersed in water, (d) the reference port is submerged in ethanol and the sensor head in water, and (e) both ports are immersed in ethanol. In addition to this, this work has been conducted with (Figure 3, black line) and without (Figure 3, blue line) the FBG connected to the output port 1 of the optical coupler.

The obtained results experimentally demonstrate the importance of including the FBG in the ring cavity configuration. As can be seen in Figure 3, when comparing black and red lines, the variations in induced losses are lower when using the FBG reflector, improving the characteristic of this interrogation system. The losses introduced into the fiber ring cavity when using the FBG ranges from 39.4–42.95 dB in cases (a) and (e), respectively. However, these losses values increase to 42.8 dB or 55.2 dB in cases (a) and (e), correspondingly, when the FBG is not connected to the fiber ring cavity configuration.

**Figure 4:**
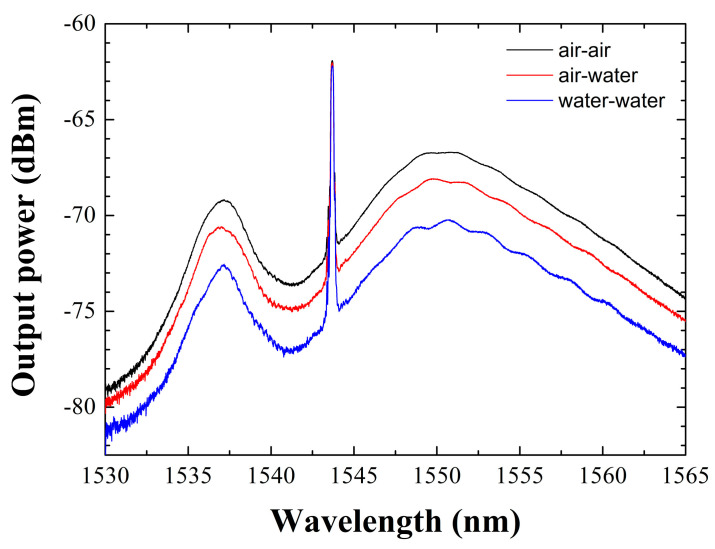
Output spectra of the fiber-ring cavity when the reference port and sensing head are in contact with air−air (black line), air-water (red line) or water-water (blue line), in that order.

For a better visualization of the results presented here, Figure 4 presents the output power spectra of the fiber cavity ring measured when the reference port and sensing head are in contact with air-air (black line), air-water (red line) or water-water (blue line), respectively. As can be observed, the peak power centered at 1543.7 nm remains constant while the amplified spontaneous emission (ASE) considerably varies in each case of the study.

**Figure 5:**
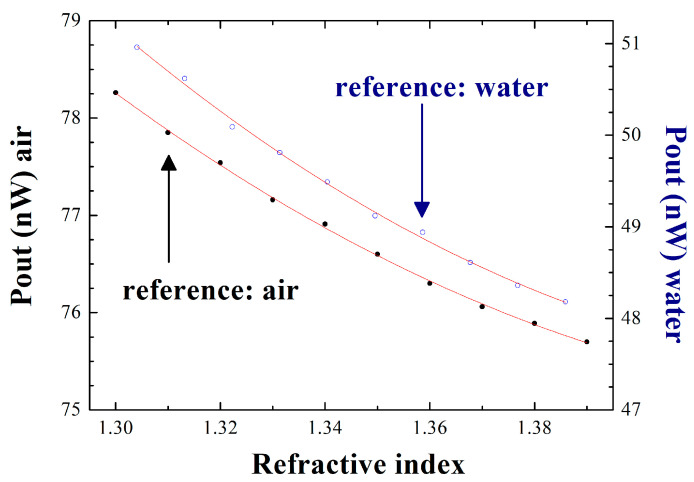
Output power levels as a function of the refractive index when the reference port-end is in contact with air (black points) or water (white points).

The effect of using air or water as a reference medium is also analyzed. Figure 5 illustrates the output power, measured using a power meter, for both situations: reference port-end is in contact with air (black points) or water (blue points), as a function of the calibrated refractive indices in which the sensor head is deep. To conduct these measurements, a calibrated refractive index kit, from the company Cargille Laboratories, is used. This refractive index liquid set includes 10 different liquids whose refractive index values are certified and, in our case, ranged from 1.300–1.390. The results presented in Figure 5 are obtained when the end of the fiber used as a sensing head is dipped one at a time into each of these samples.

In order to better quantify the amount of noise/error present in each of these two systems, a second-order polynomial approximation of the obtained measurements is performed, and the data are shown in Table 1. The adjusted R-square values obtained are 0.994 and 0.901 when the reference port-end is in contact with air or water, respectively. Also, a considerably smaller standard error in the polynomial adjustment is observed when the reference port-end is in contact with air rather than water, so both results experimentally confirm that the sensor system gives better results when the reference port-end is submerged in air. For this particular case, the accuracy of the measurements performed with this interrogation system is 0.01 nW/RIU, showing better performance than recent similar systems [11].

**Figure 6:**
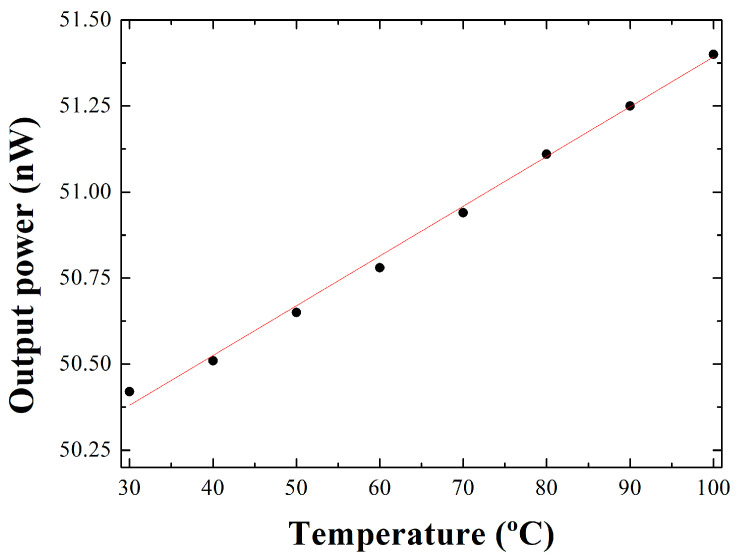
Output power levels as a function of temperature when the end of the reference port and sensor head are in contact with water.

Next, the effect of temperature variations on this sensor interrogation system was experimentally evaluated. Two studies are conducted, the first of which consists of immersing the reference port-end and the sensing head in a container of water and increasing its temperature from 30–100 °C. Figure 6 shows the output power level variations as a function of this temperature range. In the second experimental study, only one of these two optical ports is in contact with water and its behavior is analyzed as the temperature increases. The data obtained in both studies are fitted to a linear approximation, the results of which can be seen in Table 2.

Finally, the influence of the measurand-temperature cross-sensitivity effect on refractive index variations in this sensor interrogation system is analyzed. Equations (1) and (2) present the experimental results obtained for this parameter. $\Delta T_{air}$ and $\Delta T_{water}$ represent the temperature variation when the reference port is in contact with air and water, in that order. In the same way, $\Delta n_{air}$ and $\Delta n_{water}$ are the refractive index variation when the reference port-end is submerged in air and water, respectively [12].

(1) $$\frac{\Delta T_{air}}{\Delta n_{air}}=\frac{0.0145}{356.6}=4\cdot 10^{-5}$$

(2) $$\frac{\Delta T_{water}}{\Delta n_{water}}=\frac{0.006}{378.5}=1.58\cdot 10^{-5}$$

## 4. Conclusions

In this study, an interrogation system based on an erbium-doped fiber ring cavity for refractive index measurements has been presented and experimentally demonstrated. This cavity ring included a 1 × 3 coupler wherein one of the fiber output ports was used to increase the optical power of the system by means of an FBG used as a reflector and the other two output ports were used as a refractive index sensing head and reference port, respectively. This interrogation system does not need to reach the laser condition, meaning that the system is able to measure RIs using only spontaneous emission and not stimulated one. That makes this novel interrogation system a more viable solution both in terms of power and economics, due to the low level of pumping power required to perform the measurements. It has also been experimentally shown that when the medium in which the reference port is in contact with is air instead of water, the sensitivity increases and the error of the measurements is reduced. Finally, the influence of the measurand-temperature cross-sensitivity effect on refractive index variations in this sensor interrogation system is analyzed, demonstrating once again the feasibility of this interrogation system for the detection of RIs assisted by an FBG reflector.

## Figures and Tables

**Table 1:** Parameters obtained by a second-order polynomial approximation when varying RI in cases where the reference port-end is in contact with air or water. (y = a_0_ + a_1_ x + a_2_ x^2^).

Reference		a_0_	a_1_	a_2_
Air	Value	335.72	−356.61	121.19
	Standard Error	±21.18	±31.51	±11.71
	Adj. R-Square	0.994		
Water	Value	324.73	−378.5	129.17
	Standard Error	±38.9	±57.87	±21.5
	Adj. R-square	0.901		

**Table 2:** Parameters obtained by a linear approximation of the output power levels as a function of temperature. (y = a_0_ + a_1_ x).

Port Immersed in Water		a_0_	a_1_
reference port	Value	49.95	0.0145
	Standard Error	±0.021	±3.1 × 10^−4^
	Adj. R-Square	0.994	
sensor head and reference ports	Value	82.89	0.006
	Standard Error	±0.036	±5.29 × 10^−4^
	Adj. R-square	0.901	

## Data Availability

Data underlying the results presented in this paper are not publicly available at this time but may be obtained from the authors upon reasonable request.
